# Selective Blockade of TNFR1 Improves Clinical Disease and Bronchoconstriction in Experimental RSV Infection

**DOI:** 10.3390/v12101176

**Published:** 2020-10-17

**Authors:** Dorothea R. Morris, Maria Ansar, Teodora Ivanciuc, Yue Qu, Antonella Casola, Roberto P. Garofalo

**Affiliations:** 1 Division of Clinical and Experimental Immunology and Infectious Disease (CEIID), Department of Pediatrics, University of Texas Medical Branch, Galveston, TX 77555, USA; dormorri@utmb.edu (D.R.M.); teivanci@utmb.edu (T.I.); yuqu@utmb.edu (Y.Q.); ancasola@utmb.edu (A.C.); 2 Department of Microbiology and Immunology, University of Texas Medical Branch, Galveston, TX 77555, USA; maansar@utmb.edu

**Keywords:** tumor necrosis factor, TNF, TNFR1, TNFR2, respiratory syncytial virus, RSV, bronchiolitis, bronchoconstriction, neutrophils, macrophages, BALF

## Abstract

Respiratory syncytial virus (RSV) is the leading cause of bronchiolitis in infants and young children. Although some clinical studies have speculated that tumor necrosis factor (TNF)-α is a major contributor of RSV-mediated airway disease, experimental evidence remains unclear or conflicting. TNF-α initiates inflammation and cell death through two distinct receptors: TNF-receptor (TNFR)1 and TNFR2. Here we delineate the function of TNF-α by short-lasting blockade of either receptor in an experimental BALB/c mouse model of RSV infection. We demonstrate that antibody-mediated blockade of TNFR1, but not TNFR2, results in significantly improved clinical disease and bronchoconstriction as well as significant reductions of several inflammatory cytokines and chemokines, including IL-1α, IL-1β, IL-6, Ccl3, Ccl4, and Ccl5. Additionally, TNFR1 blockade was found to significantly reduce neutrophil number and activation status, consistent with the concomitant reduction of pro-neutrophilic chemokines Cxcl1 and Cxcl2. Similar protective activity was also observed when a single-dose of TNFR1 blockade was administered to mice following RSV inoculation, although this treatment resulted in improved alveolar macrophage survival rather than reduced neutrophil activation. Importantly, short-lasting blockade of TNFR1 did not affect RSV peak replication in the lung. This study suggests a potential therapeutic approach for RSV bronchiolitis based on selective blockade of TNFR1.

## 1. Introduction

Respiratory syncytial virus (RSV) is a negative-sense single stranded RNA virus of the family Pneumoviridae [1]. It is responsible for more than 33.1 million lower respiratory tract infections each year, making it the leading cause of respiratory illness in children less than five years of age. Symptoms of RSV infections range in severity from mild rhinorrhea to pneumonia [2]. The genome of RSV encodes eleven viral proteins, including two surface glycoproteins (RSV F and RSV G). Utilizing these proteins, RSV is able to invade the epithelial lining of the nasopharynx with severe cases progressing down to the small conductive airways resulting in acute bronchiolitis [3]. This involves occlusion of the bronchiolar airways due to excessive inflammation and mucus secretion, necrosis of the bronchiolar epithelium, and peribronchiolar infiltration by various leukocytes [4,5]. Several longitudinal studies have linked RSV bronchiolitis to a heightened risk of recurrent wheezing and asthma within the first 10 years of life [6,7,8,9,10]. Clinical studies analyzing cytokine mediators in the bronchoalveolar lavage fluid (BALF) of patients with RSV bronchiolitis have implicated pro-inflammatory cytokines, including tumor necrosis factor (TNF)-α, as a major contributor to disease [11,12,13]. As such, there is a growing need to develop anti-inflammatory strategies that target the “cytokine storm” associated with RSV infections, particularly those spreading to the lower respiratory tract.

TNF-α, a member of the TNF-superfamily (TNFSF), is a potent inflammatory mediator with key roles in homeostasis and disease pathogenesis. Synthesized primarily by macrophages, TNF is initially expressed as a type II transmembrane protein until cleaved to its soluble form by the metalloproteinase TNF-α-converting enzyme (TACE) [14,15,16]. Once in circulation, TNF-α mediates a variety of bioactivities by signaling through two distinct receptors: TNF-receptor (TNFR)1 and TNFR2 [17,18]. TNFR1 is widely expressed on nearly all cell types. TNFR2 is more restricted, with expression on only select lymphoid cells (CD4/CD8^+^ and regulatory T-cells), macrophages, myeloid-derived suppressor cells (MDSCs), endothelial cells, select neuronal subtypes, cardiomyocytes, and human mesenchymal stem cells [19,20,21]. As a member of the TNFR-superfamily (TNFRSF), both receptors have similar extracellular domains comprised of several cysteine-rich domains (CRD) with a pre-ligand binding assembly domain (PLAD) located at the distal end of the CRD to mediate formation of the active receptor complex [17]. The intracellular domains separate the two receptors into different subgroups of the TNFRSF. TNFR1 harbors the death domain (DD), making it a death receptor (DR). Through interactions with other DD signaling proteins, TNFR1 regulates cytotoxic signaling pathways (e.g., apoptosis and necroptosis) as well as mediate the activation of the nuclear factor kappa B (NF-κB) family and/or kinases of the MAP kinase family [14,17,22]. As with the patterns of expression, the intracellular domain of TNFR2 bears little resemblance to TNFR1. The cytoplasmic region of TNFR2 contains a short amino acid sequence that enables the recruitment of TNF receptor-associated factor (TRAF)-2 and its associated proteins (e.g., TRAF1, cellular inhibitor of apoptosis protein (cIAP)1 and cIAP2). Using these proteins, TNFR2 activates canonical and non-canonical NF-κB pathways to promote cell survival and proliferation [14,17]. These downstream properties of TNFRs make them a prime candidate for therapeutic interventions aimed to block acute and chronic inflammation.

Early studies in mouse models of RSV infection have examined the role of TNF-α in disease and viral replication using strategies to block the soluble cytokine with neutralizing antibodies and showed some improvement in disease (body weight loss), but some inconsistent affect on viral load in the lung [23,24]. More recent studies aimed to block TNF-α have used models of enhanced disease using vaccination with recombinant vaccinia-virus (rVV)-expressing RSV proteins or allergy (e.g., ovalbumin) followed by RSV challenge [25,26]. However, none of the previous studies have investigated the affect of blocking selectively each of the TNFRs in the context of experimental RSV infection. Thus, herein we delineated the function of TNF-α by administering a single dose of anti-TNFR1 or TNFR2 antibody in an experimental BALB/c mouse model of RSV infection. We estimate this single-dose to have a relatively short-lasting effect given the need for repeated administration in other studies to maintain the neutralizing activity of the antibody [27,28]. We demonstrate that this short-lasting blockade of TNFR1, but not TNFR2, results in significantly improved clinical disease and bronchoconstriction. These improvements in clinical outcomes were associated with reduction of several inflammatory cytokines and chemokines in the bronchoalveolar fluid, including IL-1β, IL-6, Ccl3, Ccl4, and Ccl5. Importantly, short-lasting blockade of either receptor did not enhance peak viral replication in the lung. Our study suggests a potential therapeutic approach for RSV bronchiolitis based on selective blockade of TNFR1.

## 2. Materials and Methods

### 2.1. Viral Infection and TNFR Blockade in Balb/c Mice

Female, 10 to 12-week-old BALB/c mice were purchased from Jackson Laboratory (Bar Harbor, ME, USA) and housed under pathogen-free conditions in the animal research facility of the University of Texas Medical Branch (UTMB), Galveston, TX, USA. All care and procedures involving mice in this study were in accordance with the recommendations in the Guide for the Care and Use of Laboratory Animals of the National Institutes of Health and UTMB institutional guidelines for animal care. A mixture of Ketamine (90–150 mg/kg) and Xylazine (7.5–16 mg/kg) was administered by intraperitoneal (IP) injection for anesthesia and euthanasia. This protocol was approved by the Institutional Animal Care and Use Committee of UTMB (protocol number 9001002J).

RSV Long strain was grown and purified as previously described [29]. Pre-infection blockade was accomplished under light anesthesia via intranasal inoculation of mice 24 h prior to infection with 80 μg of monoclonal IgG (IgG; Bio X Cell, West Lebanon, NH, USA), anti-TNFR1 (40 μg, 80 μg, 160 μg; Leinco Technologies, St. Louis, MO, USA), or anti-TNFR2 (40 μg, 80 μg, 160 μg; Bio X Cell, West Lebanon, NH, USA). All antibodies were diluted in sterile PBS to a total volume of 25 μL. At time zero, mice were intranasally inoculated with 50 μL mock inoculation of sterile PBS or with RSV diluted in PBS at a dose of 5 × 10^6^ PFU. Post-infection blockade was accomplished under light anesthesia via intransal inoculation at time zero with a 50 μL mock inoculation of sterile PBS or with RSV diluted in PBS at a dose of 5 × 10^6^ PFU. Mice were administered a second dose of anesthesia two hours post-infection (p.i.) and intranasally inoculated with 80 μg anti-TNFR1 as described above.

Animal groups were evaluated on a daily basis for weight loss a well-established parameter of clinical disease in mice. The percentage of body weight loss was plotted over time. To evaluate viral titer, serial two-fold dilutions of infected supernatants from lung homogenate were plated onto HEp-2 cells under methylcellulose overlay. Following a five day incubation, viral titer was determined by counting of plaques and calculated as PFU/gram tissue.

### 2.2. Assessment of Airway Function

Bronchoconstriction was measured in unrestrained mice using whole-body barometric plethysmography (Buxco, Troy, NY, USA) to record enhanced pauce (Penh), as previously described [29]. Penh is a dimensionless value that represents a function of the ratio of peak expiratory flow to peak inspiratory flow and a function of the timing of expiration. To establish baseline bronchoconstriction values, mice were acclimatized to the chambers for five minutes, and respiratory activity was recorded for five minutes. This protocol was designed by Buxco Electronics, and the laboratory staff was trained by the company on the use of this protocol.

### 2.3. Bronchoalveolar Lavage

At days one, two, or five p.i., mice were euthanized with an intraperitoneal injection (IP) of ketamine and xylazine followed by exsanguination via the femoral vessels. An incision was made in the trachea, through which the lungs were flushed twice with 1 mL of cold sterile PBS to obtain BALF. The chest cavity was then opened for lung collection. 100 μL of BALF was spun onto glass microscope slides and stained with H&E (Hema 3 stain, Fisher Scientific, Waltham, MA, USA) and a small aliquot was used to determine total cell counts. The remaining BALF was centrifuged and supernatants were collected and stored at −80 °C until needed for further assays.

### 2.4. Measurement of Cytokines, Chemokines, Elastase, HMGB1, and LDH Activity

Levels of cytokines and chemokines in BALF were determined at day one p.i. with a Bio-Plex Pro Mouse Group I 23-plex panel (Bio-Rad Laboratories, Hercules, CA, USA). CXCL2 was measured using a Mouse MIP-2 (CXCL2) ELISA kit (ThermoScientific, Fredrick, MD, USA). Neutrophil elastase was measured using a neutrophil elastase ELISA kit (R&D Systems, Minneapolis, MN, USA). HMBG1 and lactate dehydrogenase (LDH) were measured in BALF as markers of cell damage. Under normal conditions, HMGB1 is found within the nucleus where it stabilizes nucleosome formation and facilitates transcription factor binding. During necrosis of the cell, HMGB1 can be acetylated allowing for its secretion into the extracellular environment [30]. LDH is a stable cytoplasmic enzyme that is rapidly released upon damage to the plasma membrane [31]. HMGB1 was measured using a HMGB1 ELISA kit (IBL International GMBH, Hamburg, Germany). LDH activity was measured using the Colorimetric Lactate Dehydrogenase (LDH) Activity Assay kit (Abcam, Cambridge, UK). All kits were processed according to the manufactorers instructions. Absorbance for all microplate assays was measured on a SpectraMax 190 microplate reader (MDS Analytical Technologies, Sunnydale, CA, USA).

### 2.5. Histopathology

At days one, five or seven p.i., mice were euthanized with an intraperitoneal injection (IP) of ketamine and xylazine followed by exsanguination via the femoral vessels. Lung tissues were fixed in 10% buffered formalin followed by paraffin embedding. Multiple 4-µm longitudinal cross-sections were stained with hematoxylin and eosin (H&E). The slides were analyzed under light microscopy by a board-certified pathologist with expertise in mouse lung, who was unaware of the infection/treatment status of the animals. The grading system assigned a grade of 0–3 based on severity (0 = normal, 1 = mild, 2 = moderate, 3 = severe disease) of four different parameters: percent parenchyma involved, peribronchial lymphocytes, airway luminar exudate, and alveolar wall necrosis.

### 2.6. Statistical Analysis

Statistical analyses were performed using an ordinary one-way ANOVA followed by Tukey’s multiple comparison test and a mixed-effects model followed by Sidak’s multiple comparisons test (GraphPad Prism 8; GraphPad Software, Inc., San Diego, CA, USA). Results are expressed as mean ± SD for each experimental group unless stated otherwise and *p* ≤ 0.05 value was selected to indicate significance.

## 3. Results

### 3.1. Blockade of TNFR1, but Not TNFR2, Improves Body Weight and Bronchoconstriction

**Figure 1:**
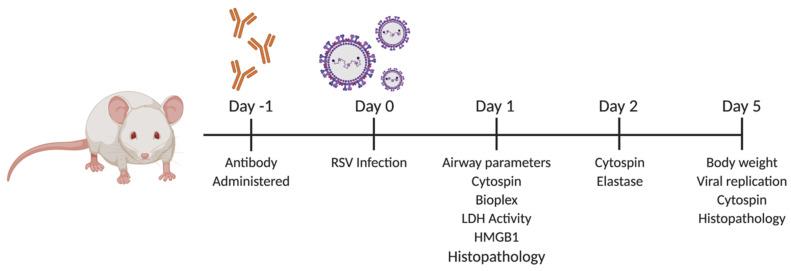
Experimental schematic of TNFR blockade for Figure 2, Figure 3, Figure 4 and Figure 5. The 10 to 12-week-old female BALB/c mice were intranasally inoculated 24 h prior to infection with 80 µg of control IgG (IgG), anti-TNFR1 (40, 80, and 160 µg), or anti-TNFR2 (40, 80, and 160 µg). All samples were diluted in sterile PBS. At day 0, mice were infected with 5 × 10^6^ PFU RSV. Following infection, mice were monitored daily for changes in clinical disease (e.g., body weight). Experiments were performed at the listed time points using lung tissue or bronchoalveolar lavage fluid (BALF) as stated elsewhere.

**Figure 2:**
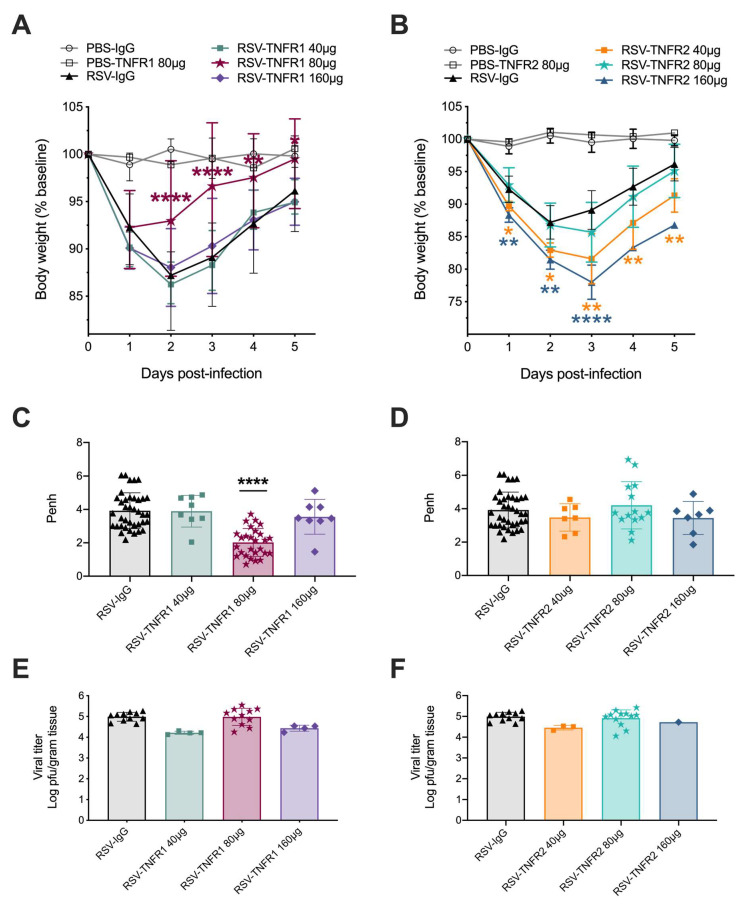
Blockade of TNFR1, but not TNFR2, improves clinical disease and bronchoconstriction in respiratory syncytial virus (RSV)-infected mice. Mice treated with varying doses of (**A**) anti-TNFR1 or (**B**) anti-TNFR2 were monitored over the five-day infection period for changes in body weight. At day one p.i., airway function, represented by baseline Penh, was assessed by plethysmography (Buxco Electronics Inc., Sharon, CT, USA) in (**C**) anti-TNFR1 or (**D**) anti-TNFR2 unrestrained mice. Lung tissue collected at day five p.i. from (**E**) anti-TNFR1 or (**F**) anti-TNFR2 mice was tested for viral titer by a viral plaque assay. Data for 40 μg and 160 μg anti-TNFR1/TNFR2 are representative of one-independent experiment. Data are pooled from four independent experiments for 80 μg anti-TNFR1 and from three independent experiments for 80 μg anti-TNFR2. All data are expressed as mean ± SD. Significant results as compared to the control RSV-IgG mice are marked with asterisks (* *p* ≤ 0.05, ** *p* ≤ 0.01, **** *p* ≤ 0.0001).

To assess the effects of TNFR blockade in the context of an RSV infection in vivo, BALB/c mice were treated intranasally with neutralizing antibodies or control IgG 24 h prior to infection as described in Figure 1. Mice were monitored over a five-day period for changes in clinical disease (e.g., body weight loss). For anti-TNFR1, RSV infected mice treated with the doses of 40 and 160 μg experienced similar peak weight loss as RSV-IgG mice (in excess of 13%) on day two and although they gradually recovered over the remaining three days they did not reach initial body weight by day 5 (Figure 2A). In contrast, mice treated with 80 μg anti-TNFR1 showed significantly less weight loss (8% peak on day one), recovered to within 3% by day three and reached their original body weight by day five. For anti-TNFR2, RSV infected mice treated with any of the three doses exhibited body weight loss similar or significantly worse than the RSV-IgG control mice (Figure 2B). In addition, the dose of 160 μg anti-TNFR2 was associated with mortality occuring at day four in two of the three RSV-infected mice. Mice inoculated with 80 μg PBS-IgG, PBS-TNFR1, or PBS-TNFR2 did not display any signs of weight-loss or disease over the five-day monitoring period, indicating that TNFR blockade alone does not lead to clinical illness in uninfected mice.

Pulmonary function following TNFR blockade was evaluated using whole-body plethysmography (Buxco Electronics, Inc., Sharon, CT, USA). For anti-TNFR1, RSV infected mice treated with the doses of 40 and 160 μg experienced similar bronchoconstriction (Penh 3.89 and 3.56, respectively) as the control RSV-IgG mice (Penh 3.92). As with the body weight loss, RSV infected mice that received the dose of 80 μg anti-TNFR1 had bronchoconstriction that was significantly improved by 49% (Penh 2.02) as compared to RSV-IgG mice (Figure 2C). For anti-TNFR2, all three doses demonstrated Penh values comparable to RSV-IgG mice (Figure 2D; Penh 3.48, 4.21, and 3.45, respectively).

Viral titers were assessed at day five p.i. by plaque assay using lung homogenate. All infected groups regardless of blockade had comparable levels of RSV replication (Figure 2E,F). Collectively, this data demonstrates that the anti-TNFR1 dose of 80 μg is the most efficacious, significantly improving RSV-induced body weight and bronchoconstriction in mice.

### 3.2. TNFR Blockade Reduces Cellular Inflammation and Cytokine Production

**Figure 3:**
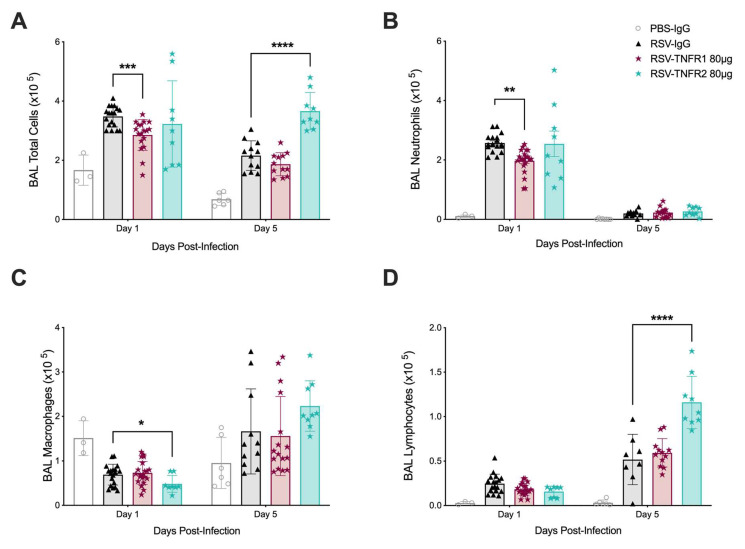
Blockade of TNFR1 reduces neutrophil number in the BALF of RSV infected mice. At day one and five p.i., BALF was collected and used to obtain (**A**) total cell counts, as well as differential cell counts consisting of (**B**) neutrophils, (**C**) macrophages, and (**D**) lymphocytes. Data for RSV-TNFR1 are pooled from at least four independent experiments for each time point. Data for RSV-TNFR2 are pooled from at least two independent experiments for each time point. All data are expressed as mean ± SD. Significant results as compared to the respective control are marked with asterisks (* for *p* ≤ 0.05, ** *p* ≤ 0.01, *** *p* ≤ 0.001, **** *p* ≤ 0.0001).

Based on the observations of improved clinical disease and airway function by 80 μg anti-TNFR1 Ab treatment and the dose of 80 μg anti-TNFR2 being the least toxic in RSV infected mice, the remainder of the study focuses on these doses. For simplicity, the dose of 80 μg anti-TNFR1 and 80 μg anti-TNFR2 will be refered to as PBS-TNFR1, PBS-TNFR2, RSV-TNFR1, or RSV-TNFR2. To determine whether TNFR blockade affected cellular composition, BALF samples were initially collected from inoculated mice at days one and five p.i. for total and differential cell counts. Total cell counts were significantly reduced by 18% at day one p.i. in the BALF of RSV-TNFR1 mice as compared to RSV-IgG mice (Figure 3A). Treatment with anti-TNFR2 did not result in any change to total cell count at day one p.i., but was significantly increased by 70% at day five p.i. as compared to RSV-IgG mice. Differential cell counts revealed this decrease in RSV-TNFR1 mice to be the result of a significant reduction by in the number of neutrophils at day one p.i. (Figure 3B ; Day 1: −23%). By day five p.i., these numbers were comparable to RSV-IgG mice. No changes in neutrophil cell counts within the BALF were appreciated for RSV-TNFR2 mice. Interestingly, macrophage cell counts were lower at day one p.i. for RSV-TNFR2 mice followed by an increasing trend in macrophage cell counts at day five p.i. (Figure 3C; Day 1: −30%, Day 5: +34%). Additionally, lymphocyte cell counts were significantly increased at day five p.i. in RSV-TNFR2 mice (Figure 3D; Day 5: +124%). No changes to macrophage or lymphocyte cell counts were appreciated in RSV-TNFR1 mice.

To examine in more detail the kinetics of cellular recruitment and specifically neutrophils, we collected BALF from RSV-TNFR1 mice at 3 h, 12 h, day one, day two, and day five p.i. for total and differential cell counts (Supplementary Materials Figure S1). Total cell counts were significantly reduced in the BALF of RSV-TNFR1 mice as early as 12 h p.i. and continued to be reduced through day five as compared to RSV-IgG mice (Figure S1A; 3 h: +13%, 12 h: −36%, Day 1: −18%, Day 2: −14%, and Day 5: −14% reduction). As described above, differential cell counts revealed this early decrease to be a result of a significant reduction in the number of neutrophils at all time points tested through day two p.i. (Figure S1B ; 3 h: −64%, 12 h: −47%, Day 1: −23%, and Day 2: −48%). Macrophage and lymphocyte cell counts within the BALF remained comparable between RSV-TNFR1 and RSV-IgG mice (Figure S1C,D). To evaluate the activation status of neutrophils, the amount of elastase released in the BALF was detected by ELISA. We found significant reductions in elastase at days one and two, mirroring the decrease in neutrophil cell counts at either day (Figure S1E).

**Figure 4:**
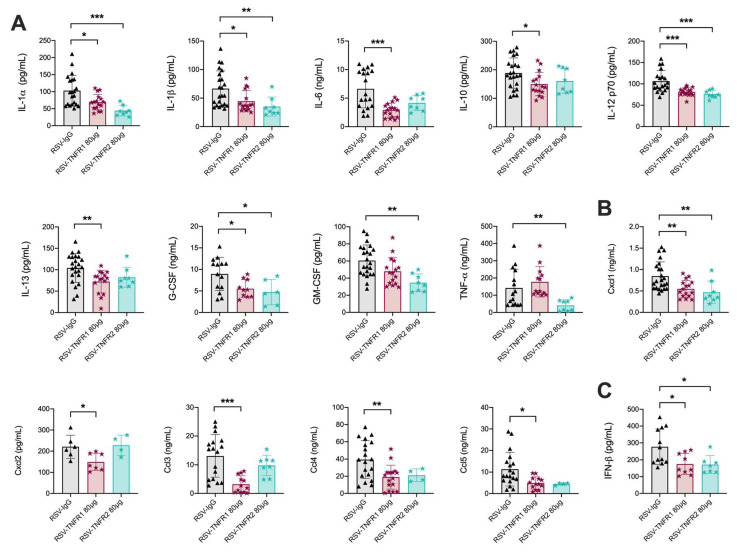
Blockade of TNFR1 or TNFR2 reduces pro-inflammatory cytokines and chemokines at day one post-infection. BALF was collected and analyzed for (**A**) cytokines and (**B**) chemokines by Bio-plex as well as (**C**) IFN-β by ELISA. Data for RSV-TNFR1 are pooled from five independent experiments. Data for RSV-TNFR2 are pooled from two independent experiments. Data are expressed as mean ± SD. Significant results as compared to the respective control are marked with asterisks (* for *p* ≤ 0.05, ** *p* ≤ 0.01, *** *p* ≤ 0.001).

We next analyzed the immunomodulatory effects of TNFR blockade during RSV infections by measuring cytokine and chemokine concentrations in the BALF via multiplex cytokine array. Cytokines and chemokines which are key to initiating pro-inflammatory responses and cellular recruitment were significantly reduced at day one p.i. in RSV-TNFR1 mice as compared to RSV-IgG, specifically IL-1α, IL-1β, IL-6, IL-10, IL-12p70, IL-13, G-CSF, Cxcl1, Cxcl2, Ccl3, Ccl4, and Ccl5 (Figure 4A,B). Suprisingly, RSV-TNFR2 mice demonstrated similar reductions in cytokines and chemokines as RSV-TNFR1 mice. Those significantly reduced include IL-1α, IL-1β, IL-12p70, G-CSF, GM-CSF, TNFα, and Cxcl1. Cytokine outcomes for RSV infected mice that received 40 or 160 μg of anti-TNFR1 or anti-TNFR2 are shown in Figures S2 and S3. Additionally, we measured IFN-β by ELISA and found it to be significantly reduced in both RSV-TNFR1 and RSV-TNFR2 mice as compared to the RSV-IgG mice (Figure 4C). All other cytokines measured for the dose of 80 μg anti-TNFR1 or anti-TNFR2 in RSV infected mice were statistically comparable to RSV-IgG mice at day on p.i. (Figures S2 and S3).

Overall, the data data demonstrates that blockade of TNFR1 prior to RSV infection effectively reduces neutrophil recruitment and activation early in infection. In contrast, blockade of TNFR2 significantly reduces the number of macrophages early in the infection while also dramatically increasing lymphocyte cell counts later in the infection. Strikingly, RSV-infected mice treated with 80 μg of either anti-TNFR1 or anti-TNFR2 had similar reductions in many of the pro-inflammatory cytokines and chemokines, with the exception of Cxcl2 and Ccl3.

### 3.3. Cell Death or Lung Pathology in RSV Infections Are Not Affected by TNFR1 Blockade

**Figure 5:**
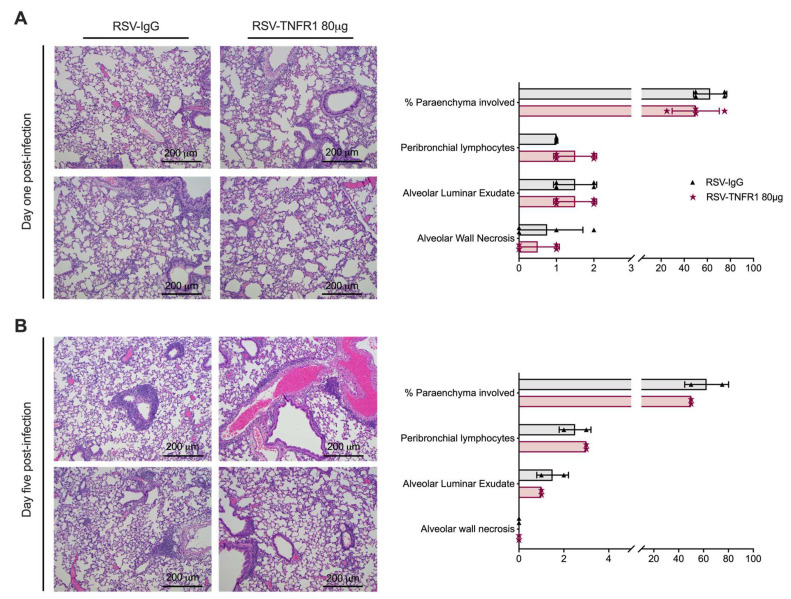
Blockade of TNFR1 at the dose of with 80 μg does not affect lung pathology in RSV infections. Lung tissue was collected at days (**A**) one and (**B**) five p.i., embedded in paraffin for sectioning and slides were stained with H&E. Pathology scores for each set are shown to the right of the lung images. Data are expressed as mean ± SD.

As described previously, TNFR1 is characterized as a death receptor with the ability to influence various aspects of tissue injury. Thus, we hypothesized that blockade of TNFR1 would result in improvements to cell damage. To evaluate this, we measured concentrations of HMGB1 and LDH in BALF and also assessed lung histopathology. Starting at 12 h, control RSV-IgG mice had a significant increase in BALF HMGB1 and LDH as compared to uninfected PBS-IgG mice. Mice in the RSV-TNFR1 group had concentrations of HMGB1 and LDH that were compareable to those in the RSV-IgG group (Figure S4). For lung pathology, lung tissue was collected at days one (Figure 5A) and five (Figure 5B) p.i., fixed in 10% formalin, and then paraffin embedded. Tissue sections were stained with H&E and graded in four categories. Overall, RSV-infected mice treated with 80 μg anti-TNFR1 had histopathologic findings similar to RSV-IgG mice at both day one and day five. Collectively, blockade of TNFR1 with a single dose of 80 μg neutralizing antibody did not modify RSV-induced cell damage or lung pathology.

### 3.4. Blockade of TNFR1 Improves Clinical Disease and Bronchoconstriction in a Model of Post-Infection Treatment

**Figure 6:**
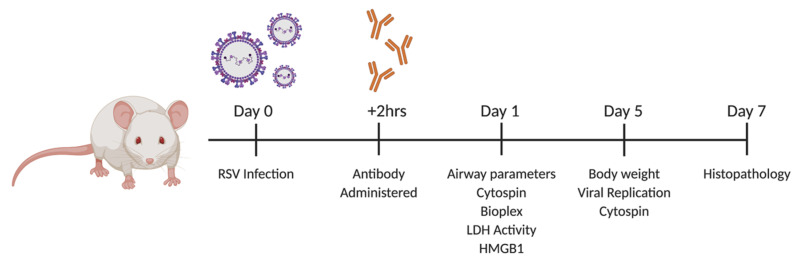
Experimental schematic of TNFR1 blockade for Figure 7, Figure 8, Figure 9, Figure 10 and Figure 11. The 10 to 12-week-old female BALB/c mice were intranasally inoculated with 80 μg of IgG or anti-TNFR1 +2 h following infection with RSV (5 × 10^6^ PFU). All samples were diluted in sterile PBS. Following infection, mice were monitored daily for changes in clinical disease (e.g., body weight). Experiments were performed at the listed time points using lung tissue or BALF as stated elsewhere.

**Figure 7:**
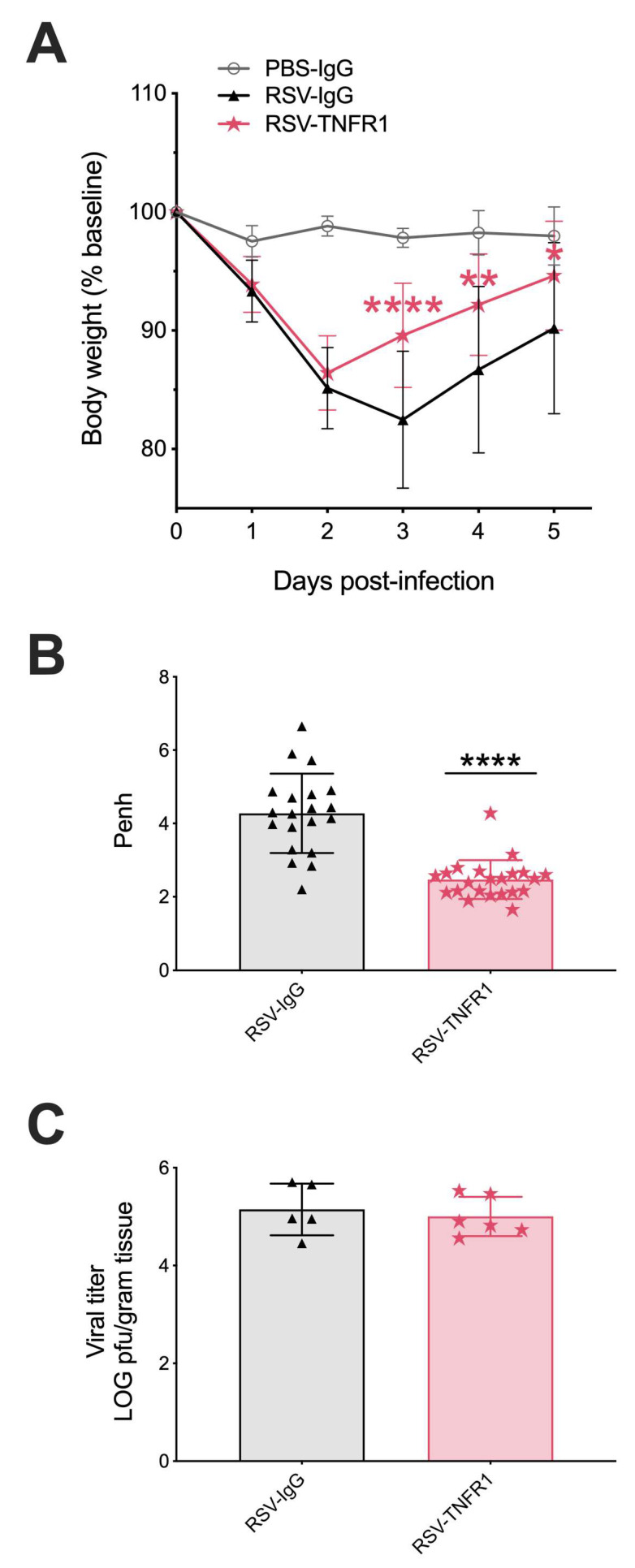
Post-infection blockade of TNFR1 improves clinical disease and bronchoconstriction in RSV infected mice. Mice were monitored over the five-day infection period for changes in (**A**) body weight as an indicator of clinical disease. On day one p.i., (**B**) bronchoconstriction, represented by baseline Penh, was assessed by unrestrained plethysmography. Lung tissue collected at day five p.i. was measured for (**C**) viral titer using lung homogenate in a viral plaque assay. Data are pooled from three independent experiments and expressed as mean ± SD. Significant results as compared to the respective control are marked with asterisks (* for *p* ≤ 0.05, ** *p* ≤ 0.01, **** *p* ≤ 0.0001).

To evaluate blockade of TNFR1 at the dose of 80 μg as a potential “treatment “option, we developed a post-infection protocol (experimental schematic shown in Figure 6). Mice were monitored over a five-day period for changes in clinical disease (e.g., body weight loss). Control RSV-IgG mice experienced peak body weight loss of 18% at day three p.i. and only recovered to within ~10% of their original body weight by day five p.i. (Figure 7A). On the other hand, RSV-TNFR1 mice experienced peak body weight loss of 14% at day two p.i., and steadily recovered to 5% of their original body weight over the remaining five days.

Pulmonary function at day one p.i. was evaluated using whole-body plethysmography. bronchoconstriction was significantly improved by 42% in RSV-TNFR1 mice (Penh of 2.47) as compared to the RSV-IgG mice (Penh of 4.27) (Figure 7B). Viral replication was assessed by plaque assay using lung homogenate from tissue collected at day five p.i. Consistent with previous results, a single dose of 80 μg anti-TNFR1 administered after viral inoculation did not affect viral replication (Figure 7C). These results indicate that 80 μg anti-TNFR1 improves clinical disease and brochial constriction in RSV infected mice in a model of post-infection treatmen.

### 3.5. Blockade of TNFR1 Improves Macrophage Cell Counts and Reduces Pro-Inflammatory Cytokines in RSV Infection

**Figure 8:**
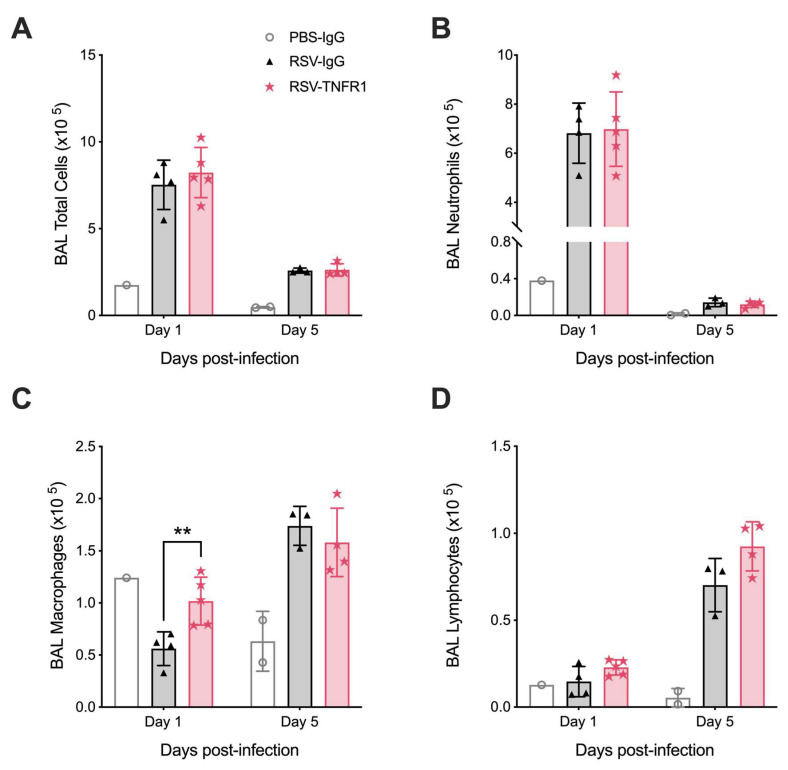
Post-infection blockade of TNFR1 improves macrophage cell counts in the BALF of RSV infected mice. At days one and five p.i., BALF was collected and used to obtain (**A**) total cell counts, as well as differential cell counts consisting of (**B**) macrophages, (**C**) lymphocytes, and (**D**) neutrophils. Data are expressed as mean ± SD and is representative of one experiment. Significant results as compared to the respective control are marked with asterisks (** *p* ≤ 0.01).

We next evaluated the effects of post-infection TNFR1 blockade on the cellular composition of the BALF at days one and five p.i. Mice in the RSV-TNFR1 group demonstrated a slight increase in total cell counts at day one p.i. with no major changes at day five p.i. (Figure 8A; Day 1: 9%). Contrary to the pre-infection model, neutrophil cell counts remained comparable to RSV-IgG at either time point (Figure 8B). This trend in total cell count at day one p.i. was shown to be the result of a significant increase in macrophage cell counts in RSV-infected mice treated with anti-TNFR1 (Figure 8C; Day 1: 81%). Lymphocyte cell counts were also found to have an increasing trend at day five p.i. as compared to RSV-IgG (Figure 8D; Day 1: 11%).

**Figure 9:**
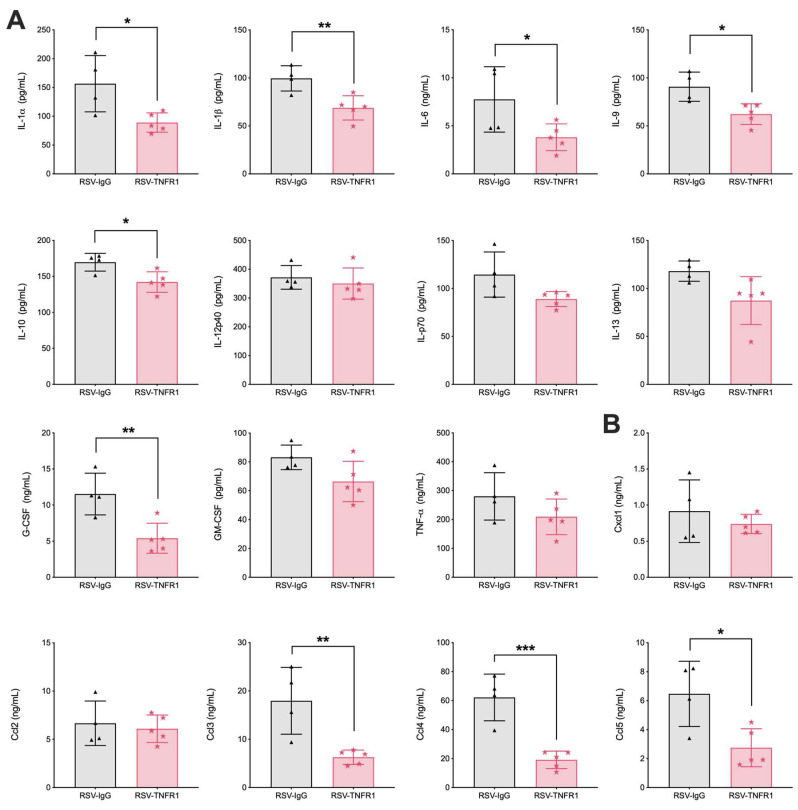
Post-infection blockade of TNFR1 reduces cytokines associated with severe RSV infections. BALF was collected at day one p.i. and analyzed for (**A**) cytokines and (**B**) chemokines by Bio-plex. Data are representative of one independent experiment. Data are expressed as mean ± SD. Significant results as compared to the respective control are marked with asterisks (* for *p* ≤ 0.05, ** *p* ≤ 0.01, *** *p* ≤ 0.001).

Furthermore, BALF was collected at day one p.i. for evaluation of cytokines and chemokines. Concentration of several pro-inflammatory cytokines were reduced, consistent with the previous model (Figure 9A; e.g., IL-1α, IL-1β, IL-6, and G-CSF). Additionally, chemokines Ccl3, Ccl4, and Ccl5 were also significantly reduced (Figure 9B). Contrary to the previous model, IL-12p70, GM-CSF, and Cxcl1 remained comparable to the RSV-IgG control. This is in agreement with the lack of neutrophil reduction noted in Figure 8B. Collectively, post-infection treatment with 80 μg anti-TNFR1 results in significantly increased macrophage cell counts and reduced pro-inflammatory cytokines with in the BALF of RSV infected mice.

### 3.6. Blockade of TNFR1 after RSV Infection Does Not Affect Cell Death or Lung Pathology

**Figure 10:**
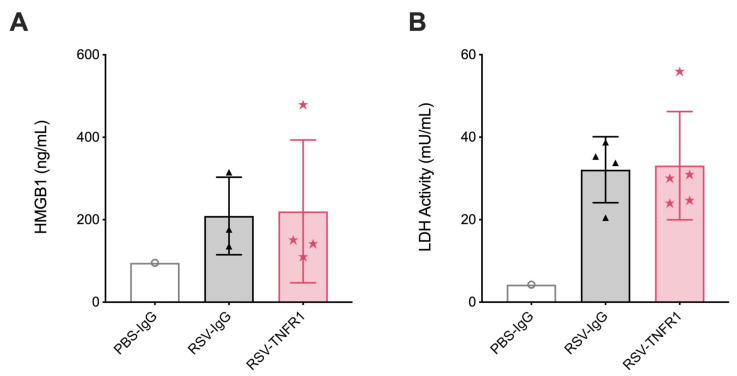
Post-infection blockade of TNFR1 does not modulate cell death in RSV infections. BALF collected at day one p.i. was analyzed for (**A**) HMGB1 and (**B**) LDH activity. Data are expressed as mean ± SD.

**Figure 11:**
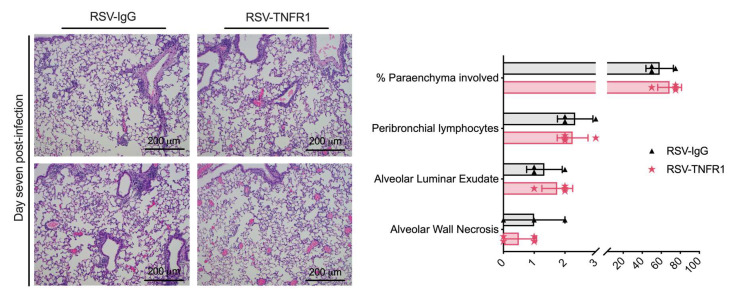
Post-infection blockade of TNFR1 does not improve lung pathology in RSV infections. Lung tissue was collected at (Left) day seven post-infection, imbedded in formalin for sectioning and slides were stained with H&E. Pathology scores shown to the right of the lung images. Data are expressed as mean ± SD.

Lung injury was evaluated by measuring HMGB1 and LDH activity in BALF, and lung pathology. Similar to the pre-treatment model, HMGB1 and LDH activity were comparable between RSV-TNFR1 mice and the RSV-IgG mice (Figure 10A,B). For lung pathology, tissue was collected at day seven p.i. and given a grade of 0–3 based on severity of disease in four categories (Figure 11). Overall, 80 μg anti-TNFR1 administered +2 h p.i. demonstrated no significant modification to lung pathology. The percent of parenchyma involvment, peribronchial lymphocyte infiltration, alveolar luminar exudate, and alveolar wall necrosis in RSV-TNFR1 mice were all comparable to the RSV-IgG mice. Collectively, a model of post-infection anti-TNFR1 inoculation does not modify cell death or lung pathology compared to RSV-IgG control.

## 4. Discussion

TNF-α is a potent inflammatory cytokine whose signaling within the respiratory tract influences a variety of biological activities, including physiological functions of the airways and cellular responses to pathogens [14]. Some studies in children have implicated TNF-α in the pathogenesis of RSV-induced bronchiolitis, but direct evidence of TNF signaling pathway in the pathogenesis of wheezing and bronchoconstriction has not been demonstrated [11,12,13,32]. Herein, we delineated the function of the TNFR-TNF-α pathway in an experimental mouse model of RSV infection by blocking either TNFR1 or TNFR2 with a single dose of neutralizing Abs. Our main findings demonstrate that mice which received an optimal dose of 80 μg of anti-TNFR1 Ab prior to viral inoculation showed significant improvements in clinical disease and airway bronchconstriction, a hallmark of RSV bronchiolitis in infants [33,34]. These same beneficial effects were also noted in our experimental “treatment” model when the Ab was administered after viral inoculation (Figure 7). Worsening clinical disease and no effect on bronchoconstriction was noted when differing doses of anti-TNFR2 were used. In agreement with our findings, other studies have found that the blockade of TNFR2 in various experimental models may exacerbate disease parameters, especially when inflammation is a key component [35,36,37,38]. Based on the results of our experiments with side-by-side use of Abs blocking TNFR1 and TNFR2, analysis of specific cytokine production, cellular inflammation in BALF, and timing of antibody administration (before or after viral inoculation), we believe that the main reason as to why TNFR1 selectively affects RSV-mediated airway bronchoconstriction is due to its known expression by airway smooth muscle (ASM) cells. Indeed, detailed expression studies have shown that TNFR1, but not TNFR2, localizes to lipid rafts of ASM [39,40,41]. Lipid rafts are cholesterol and sphingolipids enriched microdomains found within the plasma membrane of cells that aid in a number of biological functions including coordination of intracellular signaling cascades [42]. Binding of TNF-TNFR1 within lipid rafts of ASM upregulates TNFR1 localization throughout the cell as well as initiating the activation of RhoA/Rho kinase and the release of intracellular Ca^2+^. This process allows for the phosphorylation of the regulatory myosin light chain (MLC) and increased actin polymerization, perpetuating bronchoconstriction until dephosphorylation [39,43,44,45,46,47,48,49]. In TNFR1 deficient mice, these effects of TNF on ASM cells were not observed [41]. We propose that blocking TNFR1 results in improved bronchoconstriction following RSV infection by a mechanism that involves direct interference with TNF binding on ASM cells. In addition, TNF-TNFR1 binding generates intracellular reactive oxygen species (ROS) that in-turn further upregulate the wide-spread expression of TNFR1 on the cell surface [50,51]. RSV is known to significantly enhance ROS production, and previous studies in our lab have demonstrated that both controlling ROS generation and increasing antioxidant capacity in the lung leads to improved clinical parameters in mice, including body weight loss, illness score, and bronchoconstriction, in association with a significant reduction in TNF-α in the BALF [52,53]. Collectively, this suggests the TNF-TNFR1 interaction may be a key initial event that leads to airway narrowing and clinical manifestations of RSV infections, supporting interference of TNFR1 as a new potential therapeutic strategy for RSV bronchiolitis.

The cytospin analysis of pre- and post-infection blockade presented distinct BALF cellular profiles. Pre-infection blockade with 80 μg of anti-TNFR1 demonstrated reduced neutrophil number as early as 12 h p.i. (Figure S1B). This was supported by reductions in elastase (Figure S1E) and chemokines which are important for neutrophil recruitment and activation (Cxcl1 and Cxcl2) [29]. Blockade of TNFR1 at the dose of 80 μg prior to infection is likely sufficient to interfere with the strong chemotactic responses typically initiated through this receptor. These findings are in agreement with a previous report that shows reduced Cxcl1 and subsequent neutrophil recruitment in TNFR1^-/-^ mice in a model of delayed-type hypersensitivity [54]. Additionally, bone marrow derived neutrophils from TNFR1^-/-^ mice demonstrated a loss of initial neutrophil activation following stimulus with toll-like receptor ligands, suggesting that TNF-α modulates neutrophil activation in a paracrine manner [55]. RSV infected mice that received a similar dose of anti-TNFR2 demonstrated no modulation to neutrophil cell counts, but rather had significant variations in macrophage and lymphocyte cell counts throughout the infection period. Though not as well explored as TNFR1, some studies have shown that TNFR2 can modulate macrophage activity and cell death in a TNFR1 dependent manner as well as aid in the differentiation and function of regulator T-cells [17,56]. Disrupting these canonical signaling patterns between the two receptors likely produces an imbalance of cellular regulation and differentiation. Contrary to the pre-infection model, post-infection treatment with anti-TNFR1 resulted in a significant increase in macrophage cell count at day one p.i. (Figure 8C). Our lab has previously shown that RSV readily infects and kills alveolar macrophages and that their depletion leads to enhanced disease severity with minor shifts in viral replication [57]. Given that TNFR1 can initiate cell death through the death receptor, we hypothesize that this increase in macrophage number as compared to RSV-IgG mice is likely due to a delayed cell death response within this cellular subset [22].

Comparison of cytokine production from the BALF of mice treated with 80 μg anti-TNFR1 or anti-TNFR2 prior to infection with RSV yielded similar reductions, with the exception of TNF-α (Figure 4A). The TNFR2 receptor is known to upregulate TNF-α production within the macrophage population, independent of TNFR1 signaling [17]. This is in agreement with our findings of significant reductions in TNF-α production and subsequent macrophage activation following inhibition of TNFR2 (Figure 3C and Figure 4A). Similar comparisons of chemokine production demonstrated key differences that may lend possible explanations for the outcomes in clinical disease (Figure 3B). RSV-TNFR2 mice had a reduction in Cxcl1 levels, but Cxcl2, Ccl3, and Ccl4 levels were comparable to the RSV-IgG mice. Cxcl2 and its involvement in neutrophil recruitment has been linked to TNFR1 associated chemotaxis [58]. With the selective blockade of TNFR2 prior to infection, TNFR1 likely remains available for binding and initiation of signaling, as evident by the comparable levels of BALF neutrophils in RSV-TNFR2 mice (Figure 3B). Interestingly, mice treated with 40 and 160 μg anti-TNFR1 or 80 μg anti-TNFR2 demonstrated comparable Ccl3 levels to the RSV-IgG mice (Figures S2 and S3). Mice treated with 40 and 160 μg anti-TNFR2 had elevated levels of Ccl3 as compared to RSV-IgG mice. The only mice to have significantly reduced Ccl3 production were those that received 80 μg anti-TNFR1, regardless of dosing model (Figure 4B and Figure 9B). These outcomes correlate with the patterns of body weight loss in each respective group (Figure 2A,B and Figure 7A). Our lab and others have previously demonstrated a correlation between CCL3 levels and severe outcomes of disease in children infected with RSV [59,60,61].

Despite improvements in body weight loss and bronchoconstriction, TNFR1 blockade did not affect viral-induced cell death (as measured by release of HMBG1 and LDH) or lung pathology in either model. Since blocking TNFR1 did not affect viral viral load in the lung, it is conceivable that the TNF-TNFR1 pathway may contribute to clinical disease and bronchoconstriction in RSV infection by mechanisms that are either substantially less dependent or not solely dependent on the process of viral replication. The TNF-TNFR1 pathway, for example, may play an important role in amplifying or maintaining the RSV-induced cytokine storm in the lung or directly in ASM contractility, as previously mentioned. It may also be dispensable in the process of viral entry and/or replication in cells, which leads to cell death and lung damage. The lack of effect of TNFR1 blockade on RSV replication (i.e., neither reduction nor increase compared to control IgG-treated mice) in the lung has been reported in other studies in which soluble TNF-α was targeted with neutralizing antibodies [23,24,25,26].

Methods of TNF-α interference during RSV infections, whether it be blockade of TNFRs or soluble TNF-α, appear to have some similarities. In agreement with our findings, all studies have demonstrated varying levels of improvement to clinical parameters (e.g., body weight loss and illness score), irrespective of the RSV infection model [23,24,25,26]. Many studies using anti-TNF-α also noted significant reductions in neutrophil and/or macrophage activity [26,62]. Two groups have been able to demonstrate improved lung pathology. The first utilized an allergy-like model of RSV infection with rVV sensitization, designed to present a skewed Th2 response [25]. The second developed a novel anti-TNF therapy that targets only the p38 signaling axis within TNFR1 [62]. This was accomplished through identifying the appropriate sequence and developing a peptide that binds the reciprocating area within soluble TNF-α. By doing so, TNF-α is still able to initiate signaling of ERK, JNK and NFkB pathways through TNFR1. They found that this peptide blockade of the p38 pathway within TNFR1 reduces lung inflammation and, through a non-traditional assessment of viral titer, reduces RSV replication in experimentally infected mice. To our knowledge, we are the first to demonstrate significantly improved bronchoconstriction following TNFR1 blockade in an experimental mouse model of RSV infection.

Anti-TNF therapeutics have long been used for the treatment of chronic inflammatory conditions, such as rheumatoid arthritis, psoriatic arthritis, plaque psoriasis, and Crohn’s disease. Though few anti-TNFR therapeutics are currently available, there has been a recent push to develop more targeted approaches for inflammatory diseases [28,63]. In our study, we have found that a single dose of anti-TNFR1 antibody was sufficient to reduce inflammation and improve clinical parameters and bronchoconstriction in experimentally infected mice. Ideally, an RSV-infected patient would receive this treatment as a single dose through aerosolization. Supporting this notion, a recent clinical trial consisting of non-human primates and 37 healthy volunteers utilized a human anti-TNFR1 single domain antibody (GSK1995057) to successfully attenuate lung injury in an LPS-induced model [64]. In agreement with our findings, pre-treatment with GSK1995057 was able to reduce IL-1β, IL-6, and IL-8 as well as pulmonary neutrophilia. Importantly, all subjects tolerated the treatment with no adverse side affects. This lends to the potential efficacy and safety of a TNFR1 blockade therapeutic with RSV infections.

In conclusion, this study was designed to delineate the function of TNF-α by short-lasting blockade of TNFR1 or TNFR2 in an experimental BALB/c mouse model of RSV infection. We demonstrate that antibody-mediated blockade of TNFR1, but not TNFR2, results in improved clinical disease and bronchoconstriction. It also significantly reduces many cytokines and chemokines associated with severe RSV infection (i.e., IL-1α, IL-1β, IL-6, Ccl3, Ccl4, and Ccl5). Pre-infection blockade of TNFR1 was found to significantly reduce neutrophil number and activation status, consistent with the concomitant reduction of pro-neutrophilic chemokine Cxcl1 and Cxcl2. Conversely, post-infection blockade of TNFR1 significantly improves alveolar macrophage cell counts. Importantly, blockade of TNFR1 did not affect RSV peak viral replication in the lung. This study provides evidence for the use of selective TNFR1 blockade in RSV infections.

## Supplementary Materials

Supplementary materials can be found at https://www.mdpi.com/1999-4915/12/10/1176/s1. Figure S1: Time course analysis of TNFR1 blockade during RSV infection demonstrates reduced neutrophil activation early in infection; Figure S2: Cytokine and chemokine analysis of 40, 80, and 160 μg anti-TNFR1 during RSV infection; Figure S3: Cytokine and chemokine analysis of 40, 80, and 160 μg anti-TNFR2 during RSV infection; Figure S4: Blockade of TNFR1 does not modulate cell death in RSV infections.
