# Supplementary material for: Selective Blockade of TNFR1 Improves Clinical Disease and Bronchoconstriction in Experimental RSV Infection
Source: Viruses. 2020 Oct 17;12(10):1176. doi: 10.3390/v12101176 (PMC7588931; doi:10.3390/v12101176)
Supplement: Supplementary file 1 [file viruses-12-01176-s001.pdf]

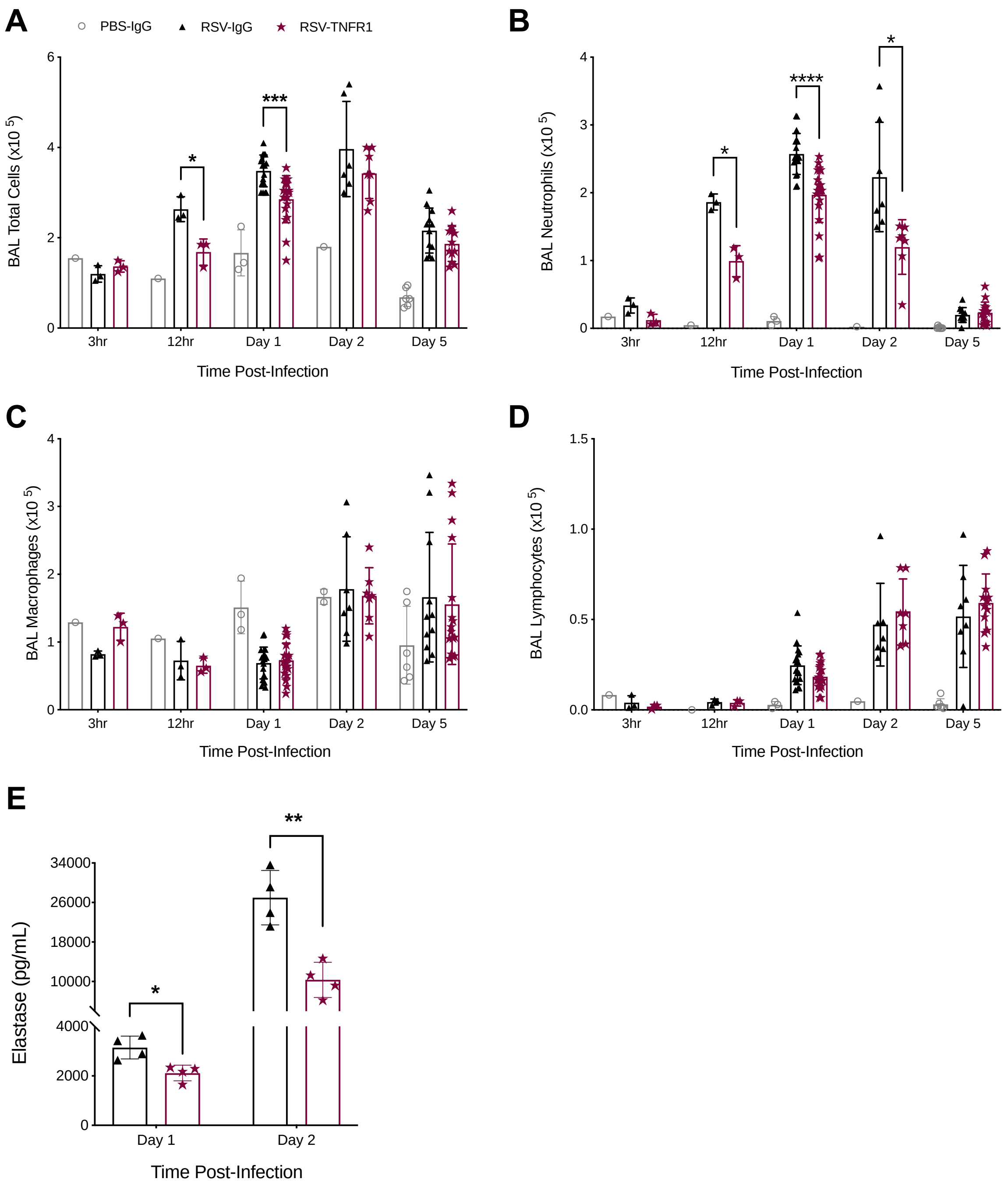

Figure S1. Time course analysis of TNFR1 blockade during RSV infection demonstrates reduced neutrophil activation early in infection. Mice were infected as described in Figure 1. BALF was collected at 3 h, 12 h, day one, day two and day five p.i. and used to obtain (A) total cell counts, as well as differential cell counts consisting of (B) neutrophils, (C) macrophages, and (D) lymphocytes. Data for 3 h and 12 h represents one independent experiment. Data for day one p.i. are pooled from five independent experiments. Data for day two p.i. are pooled from two independent experiments. Data for day five p.i. are pooled from four independent experiments. All data are expressed as mean ± SD. Significant results as compared to the respective control are marked with asterisks (* for $p \leq 0.05$, ** $p \leq 0.01$, *** $p \leq 0.001$, **** $p \leq 0.0001$).

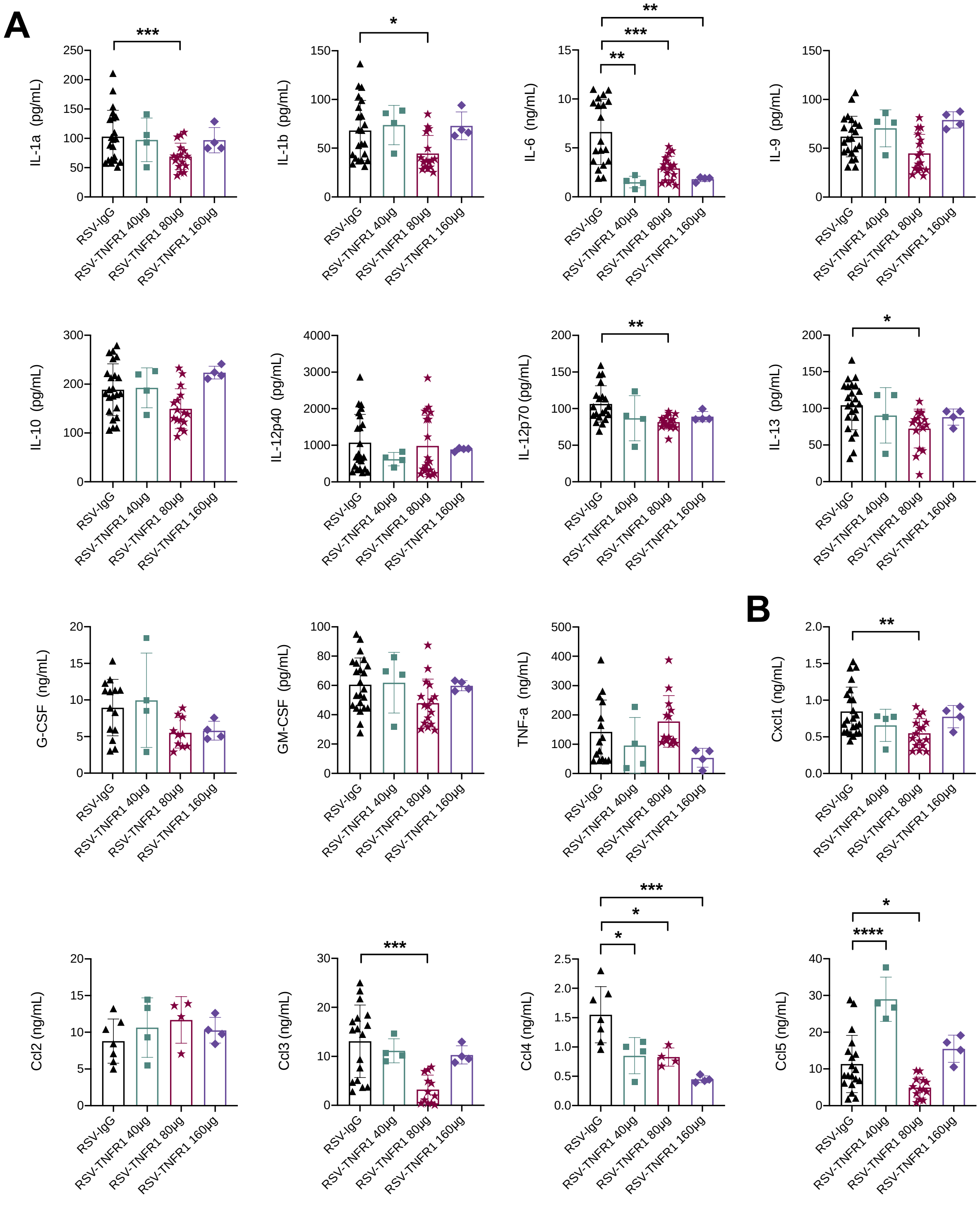

Figure S2. Cytokine and chemokine analysis of 40, 80, and 160µg anti-TNFR1 during RSV infection. Mice were infected as described in Figure 1. BALF was collected at day one p.i. and analyzed for (A) cytokines and (B) chemokines by Bio-plex as well as (C) IFN-β by ELISA. Data for 80µg RSV-TNFR1 are pooled from five independent experiments. Data for 40 and 160µg RSV-TNFR1 are from one independent experiment. Data are expressed as mean ± SD. Significant results as compared to the respective control are marked with asterisks (* for p ≤ 0.05, ** p ≤ 0.01, *** p ≤ 0.001).

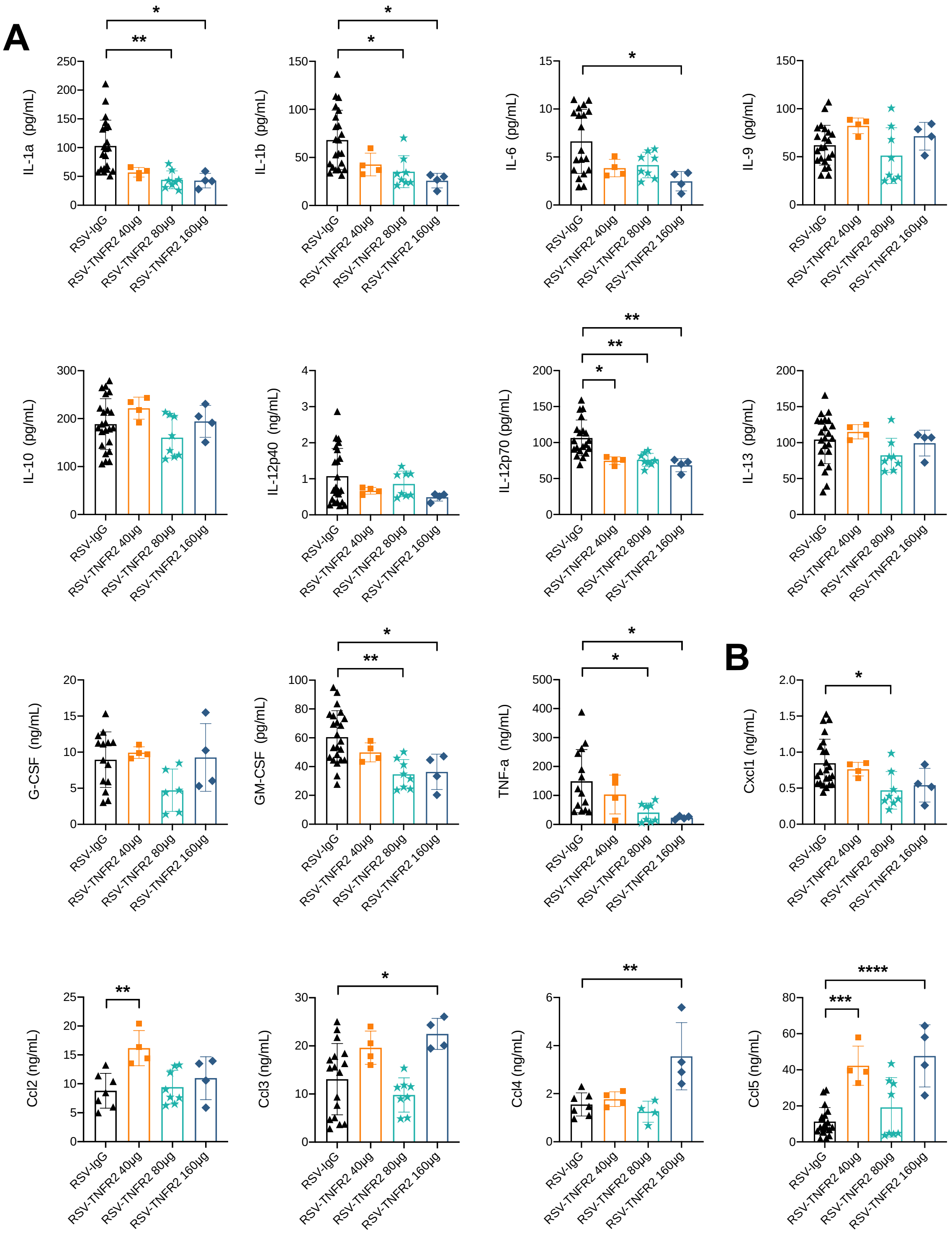

Figure S3. Cytokine and chemokine analysis of 40, 80, and 160µg anti-TNFR2 during RSV infection. Mice were infected as described in Figure 1. BALF was collected at day one p.i. and analyzed for (A) cytokines and (B) chemokines by Bio-plex as well as (C) IFN-β by ELISA. Data for 80µg RSV-TNFR2 are pooled from two independent experiments. Data for 40 and 160µg RSV-TNFR2 are from one independent experiment. Data are expressed as mean ± SD. Significant results as compared to the respective control are marked with asterisks (* for p ≤ 0.05, ** p ≤ 0.01, *** p ≤ 0.001).

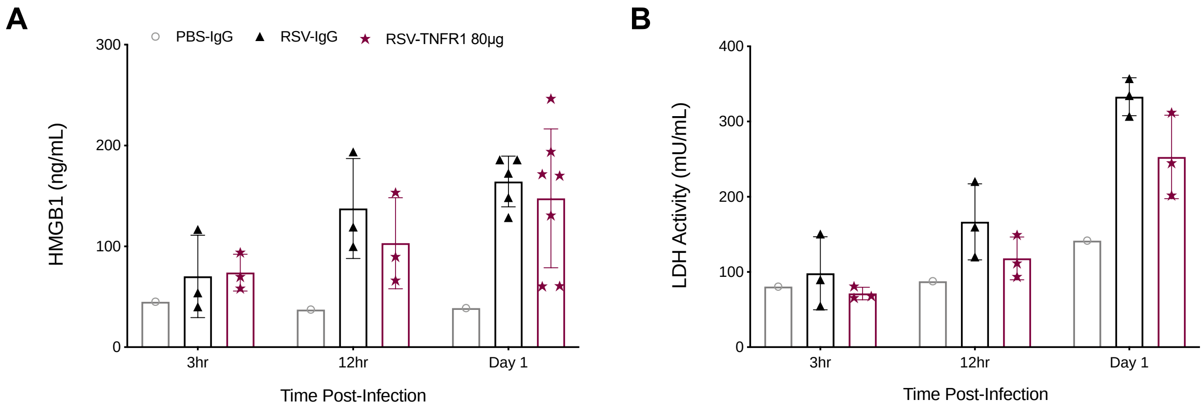

Figure S4. Blockade of TNFR1 does not modulate cell death in RSV infections. BALF collected at 3 h, 12 h, and day one p.i. was analyzed for (A) HMGB1 and (B) LDH activity. Data are expressed as mean ± SD.
